# Data Flow-Based Strategies to Improve the Interpretation and Understanding of Machine Learning Models

**DOI:** 10.3390/bioengineering11121189

**Published:** 2024-11-25

**Authors:** Michael Brimacombe

**Affiliations:** CT Children’s, University of Connecticut School of Medicine, 282 Washington Ave, Hartford, CT 06106, USA; mbrimacombe@connecticutchildrens.org

**Keywords:** A.I. artificial neural networks, data characteristics, data flow, data quality, first-stage data adjustments, orthogonalization, prediction, residual analysis, understanding

## Abstract

Data flow-based strategies that seek to improve the understanding of A.I.-based results are examined here by carefully curating and monitoring the flow of data into, for example, artificial neural networks and random forest supervised models. While these models possess structures and related fitting procedures that are highly complex, careful restriction of the data being utilized by these models can provide insight into how they interpret data structures and associated variables sets and how they are affected by differing levels of variation in the data. The goal is improving our understanding of A.I.-based supervised modeling-based results and their stability across different data sources. Some guidelines are suggested for such first-stage adjustments and related data issues.

## 1. Introduction

The opportunities presented by artificial intelligence (A.I.) in the areas of healthcare and related innovations are the focus of much interest and exciting possibilities [1,2]. The complex array of computing architectures, complex search algorithms and related programs with access to a comprehensively tagged series of massive data libraries is usually referred to as A.I. A set of modeling and clustering techniques, usually termed supervised and unsupervised machine learning [3], are usually included in the general A.I. description.

Broadly, A.I. reflects the development of computationally based machines that can perform tasks typically requiring human abilities. Typical A.I. constructs currently include (i) Generative A.I.: A.I. computing systems generating new text, images, audio or video in response to user input and using existing stored versions of these media [4]. (ii) Large language models: A.I.-based models trained on text-based data to perform language-related tasks; for example, chatbots and text generation. These are now typically available to the general public [5]. (iii) Deep learning: A form of machine learning utilizing artificial neural networks (ANNs) to recognize data patterns such as image and speech recognition [6].

At its core, A.I. is essentially an interactive computational system, interacting with various stimuli, requests and massive, complex databases. The most important aspects, at this time, are the search and optimization algorithms that allow for the identification and collation of relevant data. These procedures and related tagging/keyword search procedures have been applied to a wide variety of data [7]. All these types of data, if expressed numerically, can be further subject to a large number of functions, modeling approaches and additional algorithms, modifying and averaging whatever needs to be drawn out as a response [8]. In a sense, an A.I. algorithm searches for and whittles down the relevant information in the database each time it is requested to do so. It can also further modify these data. The algorithms search the databases according to pre-defined criteria and algorithms.

Generally speaking, A.I. is inductive, pulling in data in order to make inferences and provide results. A.I. is therefore often an open system; a set of algorithms that update constantly using a current flow of data. This is both a strength and weakness, as open systems can be manipulated by targeted flows of false or misleading data patterns [9]. The importance of data is key to the current formulations of A.I.-based approaches.

### A.I. and Healthcare Innovation

The application of A.I. to develop innovations in healthcare will no doubt be wide-ranging. The ability of the algorithms to classify patients and treatments and to do so based on existing success rates of the treatments in question may help health professionals in more efficiently treating patients. It may replace many frontline patient-related activities. The modeling of the onset of diseases in relation to lifestyle variables, clinical results, demographics, genetics, the presence of co-morbidities and secondary variables may allow for the development of highly personalized preventive medical regimes for each individual [10] that the A.I. can provide directly to patients.

As well, more clinically oriented procedures; for example, the assessment of radiology images or detailed assessment of flow cytometry-based cell counts or the detailed examination of cancer cells, may also be greatly improved as A.I. and related advancements in technology are applied [11,12]. Unsupervised learning, the clustering together of similar concepts and results, is a strong area of A.I.-related modeling and has already been applied in various healthcare settings; for example, the ranking of homogeneous groups of patients in relation to how they respond to treatments or their readmission rates. Some of these approaches have been compared to more standard statistical methods [13].

The details of many A.I. algorithms and related models are often viewed as black boxes. The issue of how to improve the understandability of such models is important. One approach is to examine how such inductive models alter as the data being processed are carefully adjusted. Given the wide-ranging nature of A.I. models, the discussion here is primarily related to predictive, supervised ANNs and random forest models.

In what follows, various data-centric strategies that seek to better understand and interpret predictive results by carefully curating the flow of data in ANNs and random forest models are reviewed and discussed. While these models possess structures and related fitting procedures that are highly complex, restriction of the data flows being utilized by these models can provide insight into how the models interpret data structures and associated sets of variables. Several approaches are examined, including residual analysis to limit variation in the data, and the use of orthogonalized variables. Various data-oriented issues affecting these models are also discussed.

## 2. Research and Predictive Modeling

The use of machine learning algorithms in the specific context of the predictive modeling of a response in relation to a set of explanatory variables, so-called supervised modeling, is an area of growing application [14]. These search algorithms, here focused on ANN and random forest models [15], have been applied in healthcare-related research and cancer research [16,17]. These models use highly flexible multi-nodal structures based on multi-nodal networks and many hidden layers [18] to generate accurate predictions.

Historically the goal of data analysis has followed the path of Occam’s Razor: keep it simple, and the most elegant and interpretable mathematical model that fits the data is a useful output. Once the mathematical model is justified by the data, and becomes representative of a theory, it can then be used to examine specific aspects of the relationships among variables, as model parameters increase or decrease. The fitted mathematical models guide not only prediction, but an understanding of the relationship or phenomenon in question. This approach underlies the development of older model-based approaches in science; for example, nonlinear regression models in toxicology and chemometrics [19], the use of mathematical models and laws in physics [20], many chemical and biochemical equations as well as most areas of scientific, engineering and medical research [21].

Newer machine learning ANN models, for example, are essentially black boxes in their structure and do not provide clear insight on how each individual variable contributes to the fitting process and the structure of the fitting process. The need for understandable results in regard to supervised data analysis, or predictive classification modeling, is a challenging requirement for data-centric modeling approaches, particularly in healthcare applications where the health of the individual will be affected. In particular, the underlying nonlinear, iterative structure of the ANN model and related fitting algorithms are not conducive to identifying the specific contribution of individual explanatory variables to predicting the response of interest.

As ANN and other machine learning models are based on observed datasets, and are often open systems, consuming flows of data in real time, there is a need to consider data-oriented strategies and methods of diagnostic assessment of model stability, convergence and meaning. The multi-nodal structure of the model and iterative fitting procedure (stochastic gradient and backward fitting algorithms [22]) allows for a very flexible modeling approach, and measures goodness of fit and predictive classification diagnostics, but also limits the understanding of how the predicted classification was obtained.

**Figure 1:**
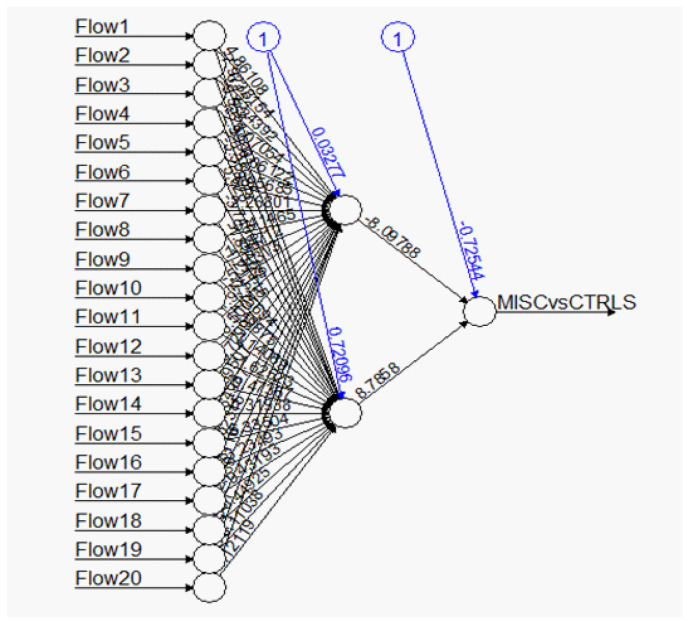
A Typical Multi-nodal ANN network model graphic linking input variables with a binary output.

A typical example of an artificial neural network-related output is shown in Figure 1, and shows the difficulty of clearly interpreting the relative importance of each variable in a set of flow cytometry variables used to predict an outcome of MIS-C versus Control patients. Note that the ANN model applies to the prediction of binary outcomes.

As well, the convergence paths of such ANN models can be quite unstable, often converging and de-converging as the data are processed and the model allowed to become even more complex with additional hidden layers and structures [23]. Is this “deep learning”, a new type of knowledge of the scientific or medical response being studied, or simply the fitful convergence patterns of an overly complex, data-based, quantitative model? These remain open issues for researchers to consider [24].

### 2.1. ANN Models

The application of data-centric machine learning approaches to the development of predictive models in medical and scientific research has recently grown substantially as a major component of A.I.-based data science, providing an alternative to the pre-experimental perspective of population-based probability models and statistically designed experiments [1,2,3].

In the next few years, if not already, much standard quantitative analysis in medicine and both pediatric and overall health studies may be augmented by data-centric approaches. Unsupervised ANN modeling, essentially cluster analysis, has been shown to be very useful [25,26]. However, while accurate in large datasets, supervised ANN predictive classification models may be only moderately predictive in small-to-moderate sample sizes and their results are not generalizable [27].

While predictive in many cases, such highly complex and flexible structures may not give rise to an understanding of the underlying scientific or medical processes underlying the prediction. Many basic relationships among biological and chemical variables have been understood using much simpler and more interpretable probability-based models [28]. Simple constructs such as generalized linear models track the contribution of individual variables in relation to the overall variation in the response of interest, often with ANOVA tables providing guidance. The perspective taken here is that predictive models in general should balance improved understanding where possible, with high levels of prediction.

### 2.2. Model Assessments and Empirical Probability

The application of supervised ANN models, even if they are highly complicated constructs that are difficult to interpret, require diagnostic measures to assess how accurately the training model predicts the response of interest in the testing data for a set of explanatory variables. To achieve this, a type of empirical probability-based approach has been developed [29].

For example, the application of a supervised ANN classification model will yield the output of a logistic function, often re-named the sigmoidal function, giving a value between 0 and 1. If the data are for a group of individuals, each individual receives a value and each value is interpreted in relation to a selected cut-off value, usually 0.5. If the value is above 0.5, the individual is predicted to have the response of interest, and if the value is below, they are predicted not to have the response.

This is not performed for the data as a whole. That is essentially statistical logistic regression if the sample is viewed as selected randomly from a larger population. Rather, in the application of the supervised ANN model to the dataset alone, however collected, the data are first randomly split into training and testing components. The model is fit to the “training” component and used to predict the response of interest (0/1) in the “testing” component of the data. This allows for the development of a set of classification diagnostics similar to those of logistic regression: ROC curve, AUC, sensitivity, specificity, false positives, false negatives, overall classification accuracy. As well, this random splitting process might be repeated 10 times to account for random variation in the training/testing data selection and provide useful median values and ranges for these diagnostic values.

This use of data-based, empirical probability [29] makes clear the data-based aspect of supervised machine learning. How to extend the meaning of machine learning results to other contexts beyond the specific dataset at hand is a challenge. It is necessary to render A.I.-based results relevant to broader settings. It is also necessary to continually update data-centric A.I. results over time, updating the analyses to account for the constantly changing databases.

### 2.3. First Stage Data Adjustments in Predictive Models

The data-centric nature of A.I. and machine learning models requires careful curation and assessment of the data themselves. Often, apart from the response variable, a set of potential explanatory variables thought immediately relevant to predictive modeling is usually the primary focus. Secondary variables defining the broad context of the data; for example, demographics, age, gender and other mediating variables, may also be included in the development of the predictive model.

In many current applications of machine learning models; for example, supervised ANNs and random forest models, the practice is usually to include all relevant variables in the model and focus directly on the predictive accuracy of the resulting complex fitted model [30]. From a statistical perspective, the use of such data and sets of variables without careful adjustment will often lead to a large amount of extraneous variation, especially when dealing with the data drawn from non-experimental settings. This affects the accuracy and convergence of the model. Defining secondary and mediating variables are typically essential to the interpretation of such information [31].

**Figure 2:**
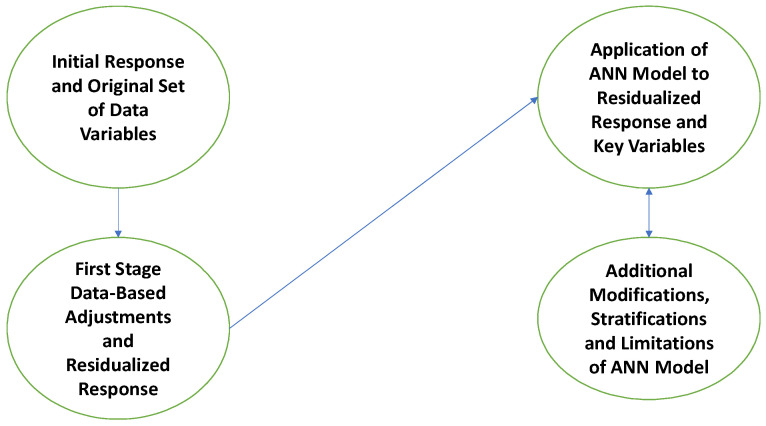
First-stage data flow adjustments.

Adjusting the dataset in question for variation due to presence of secondary or mediating variables will often allow for subsequent comparison to datasets drawn from different sources: hospitals, marketing databases, government healthcare databases, similar healthcare databases across a set of different regions or countries. In order to apply across various databases, such initial adjustments should be kept as simple as possible, typically linear in nature. See Figure 2.

In statistical models that use population-based probability concepts, the variation due to secondary variables is often controlled by restricting the design of the study or experiment generating the data. When this is not possible; for example, when analyzing previously collected large electronic databases, it is often necessary to control for the variation due to secondary variables by including them in the model, typically a linear model as control or mediating variables [31]. This is effectively similar to modeling the response using the secondary variables and then modeling the resulting residual vector using the variables of interest.

Due to the more complex and nonlinear nature of ANNs and random forest models, it may be prudent to use secondary variables as an initial set of mediating variables, applying a simple linear model that links the response to these variables and removing the variation in the response due to these variables. The resulting residuals can then be modeled using the supervised ANN model in relation to the primary variables of interest.

An alternative, but related approach is to use propensity scores [32] as a variable in the predictive ANN model where the propensity score replaces the set of secondary or mediating variables. The use of propensity scores for binary outcome-based supervised ANN predictive models. In particular, stratification across levels of the propensity score variable and inclusion of the propensity score variable in the model can be compared and examined.

In the case of random forest models applied to a continuous response, the response is first projected onto the linear model space defined by the secondary variables of interest. The residual vector is then used in the random forest model, replacing the original response vector. This reduces extraneous variation, allowing for a more accurate assessment of the relationship between the response and the variables of interest.

Given the wide-ranging application of ANN models [10,11,12], the data-based strategies briefly reviewed here may improve the ability of a wide array of medical researchers, public health professionals and trained clinicians who use the output of such models to better interpret their findings. It will also allow for similar sets of variables to be modeled and compared more easily across differing data collection sites; for example, hospitals or regional healthcare systems, by controlling for similar sets of secondary variables, applying initial data transformations to better standardize data prior to analysis.

## 3. Residual Analysis: Mediating Variables

In many settings, usually observational studies based on datasets with limited pre-experimental study design, demographic and other contextual variables can be adjusted for in the analysis to improve comparability of, for example, treatment and control cohorts in relation to primary variables of interest [31].

When using supervised ANNs to model the relationship between say, the outcome *y* and a set of explanatory variables $x_{1},\ldots ,x_{p}$, an initial linear adjustment to the response can easily be carried out, effectively removing the variation in the response due to secondary and demographic variables. Letting the secondary variables be denoted $f_{1},\ldots ,f_{m}$, an initial linear model can be used to model *y* in terms of these variables. Typically a least squares fit can be used where $F=[f_{1},\ldots ,f_{m}]$ is an *m* by *n* data matrix and the least squares estimate of *y* is given by

(1) $$\hat{y}={\left(F^{\prime }F\right)}^{-1}F^{\prime }y$$

and the residual vector is given by $\hat{\varepsilon }=\left(y-\hat{y}\right)$. The variation in the response *y* not due to these secondary variables will be contained in $\hat{\varepsilon }$. This should be less than the initial level of variation in the overall dataset and allows for the removal of demographic and secondary effects6related variation in a controlled, interpretable manner.

### 3.1. Residualized ANN Model

The ANN model is typically used to model or classify a binary output $y_{i}$ in relation to a set of explanatory variables $X=[x_{1},\ldots ,x_{n}]$. As with logistic regression models, the logistic function can be used to link a continuous input to an output lying between 0 and 1 at each nodal value. This can be written as follows:

(2) $$\eta \left(x\right)=\frac{1}{1+e^{-x}}$$

for x>0. The structure of the ANN model sets up layers of neurons, each of which takes on values based on the η(x) function. This set of $\eta_{i}\left(x\right)$ values can be seen as generating a vector of outcomes of nodal values for each hidden layer, $h_{j}$, i=1,…,m, as the fitting algorithm is applied. Write this as $a_{i}^{\prime }=\left(a_{1},\ldots ,a_{n}\right)$.

The fitting procedure is iterative, with the results of each layer passed to the next layer, after being adjusted by selected weights (W) and biases (b) at each stage, so-called fitting parameters. The number of parameters at each stage far exceeds the number of explanatory variables at a given stage (n) as they include all parameters previously calculated at each hidden layer nodes. These are re-adjusted as the fitting procedure continues [33].

This can be expressed generally as

(3) η(Wa+b)

with the $i^{th}$ nodal value $a_{i}$ for the next layer given by

(4) $$=\eta (\sum_{j}w_{ij}a_{j}+b_{i})$$

These iterative calculations, for example, in the third hidden layer of the ANN model, are of the form

(5) $$\eta (W_{3}\left[\eta \left(W_{2}a+b_{2}\right)\right]+b_{3})$$

To fit the model to the observed data, $y(x_{i})$, a cost function is typically defined based on a measure of distance to be minimized between fitted model and observed outcome, for example:

(6) $$Cost\left(W_{1},W_{2},W_{3},b_{1},b_{2},b_{3}\right)=\sum_{i}||y\left(x_{i}\right)-\eta \left(W_{3}\left[\eta \left(W_{2}a+b_{2}\right)\right]+b_{3}\right){\left|\right|}^{2}$$

This expression is a function of the parameters with the data y(x) known. The training of the network is essentially the selecting of parameter values to minimize the Cost(·) function. Again, as the fitting or “training” procedure is expanded to increase the number of hidden layers, the earlier parameters are modified via back-propagation with convergence supported by applying stochastic gradient descent methods [3].

The resulting ANN model based on the residual vectors will use as a basic component

(7) $$\eta \left(\hat{\varepsilon }\right)=\frac{1}{1+e^{-\hat{\varepsilon }}}$$

When comparing ANN models built using different datasets; for example, studies conducted on different hospital databases, the resulting ANN fitted models can be better compared if similar initial linear adjustments are carried out, adjusting for difference demographics, for example. For example, when comparing two such models, the analysis will be carried out using residual vectors, adjusted for the same set of demographics, improving comparability of the analyses. The ANN models will then use as basic respective components

(8) $$\begin{array}{cc} \eta ({\hat{\varepsilon }}_{1}) & =\frac{1}{1+e^{-{\hat{\varepsilon }}_{1}}} \end{array}$$

(9) $$\begin{array}{cc} \eta ({\hat{\varepsilon }}_{2}) & =\frac{1}{1+e^{-\hat{\varepsilon_{2}}}} \end{array}$$

where $\hat{\varepsilon_{i}}=\left(y_{i}-{\left(F_{i}^{\prime }F_{i}\right)}^{-1}F_{i}^{\prime }y_{i}\right)$. Predictive classification diagnostics can then be used to compare the fit of the ANN models of interest.

Such first-stage adjustments can also be carried out and compared to ANN models built using the entire dataset, giving some measure of the importance of context. If the first-stage adjusted analysis does not alter from the initial overall model, this is evidence that the results found are stable across the levels of the secondary variables and they can safely be disregarded. This measure of model robustness is studied in detail elsewhere.

### 3.2. Small Sample Accuracy

Note that the need for these types of adjustments grows as the dataset has lower sample size or relatively high levels of variation. In such settings, ANN models may not provide useful summaries. For example, Table 1 gives the results of an example simulation study (data available upon request) examining the relationship of a neurologic outcome to a set of explanatory variables. The study had a moderate sample size and high levels of variation. The predictive accuracy here is limited and does not improve when the model is allowed to become more complex.

Removing the effects of secondary variables prior to final analysis may improve and stabilize such smaller sample analyses.

Note that, if the use of such first-stage residual analysis results in a lowering of the variation in the response, it would be expected that the supervised ANN model will require a lower number of hidden layers, and that the complexity of the ANN model will be reduced.

## 4. Orthogonalization and Data Cloud Descriptions

In cases where secondary variables are not necessarily a potential source of extraneous variation; for example, in collections of data variables that are entirely genetic expression values, supervised ANN classification models may be both more predictive and converge more rapidly if an initial transformation to orthogonal axes is carried out. The use of orthogonal axes limits the effects of correlation among the data vectors on the convergence process while not altering the basic information-related properties of the ANN model.

This orthogonalization of the set of data vectors implies representing the initial data vectors, all of them, as a data point cloud in relation to a new set of coordinate axes. Geometrically this broadens the span of the coordinate axis within which the data points are expressed. The method of principal components [34] is the most direct and interpretable approach to do this. The use of an orthogonal set of axes allows for a wider linear span in which to obtain an initial fit, without the correlation of the data elements complicating the fitting algorithm. The initial data vectors are re-expressed in terms of the new, orthogonal axes.

### Geometry

Sample principal components can be interpreted geometrically. Assuming the joint distribution of the original (centered) data variables $x=(x_{1},\ldots ,x_{p})$ can be viewed as having an approximate p—dimensional multivariate normal distribution of the form N(0,Σ), then most of the data will lie in a hyper-ellipsoid region whose contours can be approximated as

$${\left(x-\bar{x}\right)}^{\prime }S^{-1}\left(x-\bar{x}\right)=c$$

where the axes of the ellipsoid correspond to the eigenvectors of *S* and the length of the axes are proportional to the square root of each eigenvalue of *S*.

The goal here is to re-scale the original correlated data vectors into a set of uncorrelated data vectors, the principal components. Since the principal components are a linear combination of the original data vectors, they can also be viewed as normally distributed, centered, variables, $y=e^{\prime }\left(x-\mu \right)$ where *e* are the eigenvalues. Once the resulting *p* sample principal components are obtained and viewed as transformed data, these new data vectors can be viewed as being plotted against the *p* eigenvectors of *S*, which provide an orthogonal basis. The resulting hyperellipsoid then becomes a p—dimensional sphere centered at the value $\bar{x}=\left({\bar{x}}_{1},\ldots ,{\bar{x}}_{p}\right)$. The sample principal components can be viewed as the result of translating the original origin to the sample mean and rotating the coordinate axes until they pass through the scatter of the original data cloud in the directions of maximum variance [34].

This may provide a useful first-stage adjustment in small-to-moderate sample sizes. It does not affect the overall information in the data or the interpretation of the overall model–data combination since the model, with or without the transformation, provides a black box-based interpretation.

## 5. Data Structures and Limitations

Additional data quality considerations arise when examining ANNs and other supervised models, looking to interpret the data in relation to a broader context. This typically reflects whether the dataset is representative in relation to a background population; for example, a network of hospitals or set of census tracts. These considerations include probability-based weighting of survey data [35], the use of censoring in time to event analysis [36] and the need to address data structures and possible latent variables [37] in the dataset. Also, the presence of impactful secondary variables affecting the predictive accuracy of the supervised ANN or random forest model may require stratification of the machine learning model within the levels of the secondary variable.

For example, in the setting of statistical modeling, when there are more variables than subjects, p>n, standard methods such as least squares or maximum likelihood do not converge and are not useful. The Lasso [38] or moving-window approach is often used to generate a series of smaller fitted models with n>p in each.

This type of strategy to model fitting can be used in the setting of machine learning models [39]. Both ANNs and random forest models can be applied in this manner, generating a series of predictive models and related predictive classification diagnostics. In settings wherein the number of parameters is greater than the sample size, the model is not immediately identifiable. To deal with this, a sparsity restriction might be placed on the set of nonlinear models being considered:

$$\begin{array}{c} y_{i}=ML\left(\left\{x_{ij}\right\}\right)+\varepsilon_{i}\\ \left\{x_{j}\right\}\in x_{1},x_{2,}\cdots x_{p} \end{array}$$

Note that the effect of applying the sparsity window to estimation of such an iterative, non-linear model may substantially alter aspects of the model for some of the windows being considered. It may also re-weight the correlation structures present in the data.

The development of analytic strategies for data-centric machine learning supervised models requires careful assessment of the flow of data, the definition of data variables and how these variables are scaled.

Some additional approaches related to interpretation and data curation are relevant.

### 5.1. Modifying Predictive Accuracy

Both ANNs and logistic regression models yield, as an output for each subject, a value in (0,1). These give a predicted classification value of Yes or a No according to whether the value is greater than a chosen cutoff, typically p=0.5. In logistic regression applications where a population-based perspective is taken, if the response of interest is rare, the cutoff may be lowered to 0.3 or 0.2. This sometimes gives better model sensitivity and specificity.

This type of consideration may also apply to ANN models if the researcher considers the response of interest to be rare in the data, especially in the testing data. If comparisons are to be made between a logistic model and an ANN model, the cutoff should be set at the same level. Note that in regard to the ANN model, the random splitting of the overall dataset into training and testing sets is often set at 50/50 or 70/30 levels.

### 5.2. Stratification Based on Mediating Variables

When modeling with machine learning approaches, if the overall prediction classification result alters when additional, secondary mediating variables are added to the model, then running the original ANN model within data categories defined by the secondary, mediating variables [31,40] and examining the stability of the resulting predictive classification may be necessary. These secondary variables often include age group, gender, region, types of comorbidities or other study-related factors. This type of stratification is essential to understanding the appropriate context for the observed patterns and predictions in the dataset.

In the case of machine learning models (ML) such as supervised neural networks and random forests, we might use a similar simplified general notation:

y=ML(X,β)+ε

is the overall model and

$$\begin{array}{cc} y_{1} & =ML_{1}\left(X_{1},\beta_{1}\right)+\varepsilon_{1}\\ y_{2} & =ML_{2}\left(X_{2},\beta_{2}\right)+\varepsilon_{2} \end{array}$$

where each $ML_{i}\left(\cdot \right)$ is fit to the $i^{th}$ sub-group.

As ML models are to some extent black boxes, validated by comparing classification results achieved in relation to how well the training data-based models predict the testing portion of each $X_{i}$ dataset, “differences” between the overall modeling result and the conditional within-strata results can be assessed by comparing classification diagnostics (for example, ROC, AUC) for both overall and within sub-group models.

### 5.3. Probability Weighting and Censoring

Probability-based adjustments and definitions (weighting, censoring, random effects etc.) are standard elements of frequentist assessment and interpretation of large-scale survey data [34,35,36]. These may need to be re-developed or incorporated directly into the context of data-centric machine learning methods in order to appropriately interpret the dataset itself and related data-centric inferences. Subsets of the data with probability of selection of 1/100 do not have the same degree of representation as those drawn with 1/10,000 probability. This is often the case in large survey data drawn from various cities and their census tracts with varying population densities.

There are some after-the-fact adjustments available for such considerations. For example, propensity scores [32], variables generated for a set of demographic variables to adjust for sampling intensity differences across sub-groups, may be incorporated into the neural network modeling scheme, or used to stratify the data. At a minimum, such strata should weight the modeling of both training and testing groups to improve comparability.

Censored data [36] are also an issue. For example, in the study of disease onset, the onset for some individuals is censored by the end of the study. This is usually dealt with by incorporating a probability-based adjustment to the model reflecting this fact and this becomes part of the overall likelihood function for the study. It may be possible to re-weight specific elements of the neural network in a similar manner.

Note that application of such data modifications in the ANN context should be justified in both testing and training samples.

### 5.4. Data Structures in Predictive Models

Data structures are important. But often their structure is not particularly a focus in population-based statistical inference. The Simpson’s paradox and other structures reflective of latent or secondary variables can seriously affect the accuracy and relevance of inference [37,41]. Population genetic data also have specific data structures affecting interpretation [42]. The detection of anomalous data structures may be even more important in large datasets where they may occur more frequently.

Other possible data structure-based challenges to interpretation and comparison include non-random missingness in the data [43]. If this is not occurring at random, it may affect all these different analytic approaches, typically by creating bias in the data themselves. This speaks again to the importance of how data are collected and the need for quality design and understanding, whether the data are being generated from a randomized clinical trial or simply being collected from large, existing electronic databases with unknown data structures.

Note that all the methods considered here are prey to potential bias in the data, when the data are not collected in an unbiased and representative manner. There remains an important place for the further development of quality randomized and designed methods of data collection for machine learning applications.

When applying simple linear models to the analysis of data, the ANOVA table is available and parses out the relative contribution of each variable in relation to how much of the overall variation is explained by its presence in the analysis of generalized linear models. This is performed via orthogonal projection and, in the cases of normally distributed response, in terms of uncorrelated ANOVA squared-length components with test statistics in the form of signal-to-noise ratios [41]. The information value of the ANOVA table and related confidence/credible intervals is difficult to numerically quantify, but they are often highly informative and this information is not available when applying supervised ANN models for prediction.

### 5.5. Defining Variables for Analysis

All models, statistical and supervised machine learning, require experimental context and measurements. Specific hypotheses make the analysis easier and more focused, but often prediction is the primary goal. As many applications of machine learning are somewhat new, there is sometimes a need for justification of variable selection and the scales on which the variables of interest are defined. Variable selection should obviously reflect the science or medicine involved and the specific aims or hypotheses of interest.

Machine learning methods are heavily data-centric, with the ANN model using a flexible multi-nodal network-based mathematical structure that is fit to the training portion of the data and then used to predict the response in the testing portion of the data. This is performed using random splits of the existing dataset into training and testing portions. The issues of data variable definition and scaling are both flexible and very important in these settings. Which variables to include in the ANN model, and how to give these variables numerical representation, is often non-trivial. Related to this are issues of model convergence, accuracy and complexity in the form of hidden layers.

Some typical questions that arise relevant to the variable selection process:

- Why were these particular secondary variables selected?
- Why were these specific age intervals chosen?
- What justifies non-standard re-numbering schemes for count variables such as Year?
- How is the selection of 100 hidden layers in the ANN model justified? How much variation in predictive accuracy was there going from 50 hidden layers to 100?

These definitions of context, variables and scales of measurement require insights from the medicine or science of interest. Putting all possible variables in a model and hoping for the best, a common practice in many machine learning contexts, is potentially very misleading and will incorporate large amounts of irrelevant variation into the modeling process. This should be avoided. The goal is understanding.

#### Example

Consider the modeling of cancer incidence. Here the numerator = count per month and the denominator = population per month, resulting in monthly incidence = count/population. In some applications, the count and population variables are defined as separate variables in ANN models. This is potentially misleading. For example, if the count variable has two entries of, say, 15, these would be equal on the count scale. But if one value corresponds to a respective monthly population of 100 and the other to a monthly population of 100,000, the two values of 15 represent very different incidence values. It is misleading and incorrect to view count and population as separate variables in a study on incidence.

### 5.6. Database Quality

A.I., as currently structured, heavily depends on access to underlying databases, upon which search algorithms can be applied. The quality of the data collections must be curated and maintained carefully for researchers and others to have confidence in the results of the A.I. Consistency in variable definition and measurement approaches must be maintained. Developing more understandable A.I. procedures requires stability of the data structures and high quality of the data themselves. This is often an expensive and time-consuming effort. Small numbers of missing data, outliers and poorly defined variables can throw off the complex search algorithms defining A.I., though they have been.

Datasets, the individuals themselves whose data are collected in the database, the variables and measurements collected, images, e-mails, texts and computational architectures, may lose relevance over time, affecting the comparability of A.I.-based predictions.

## 6. Discussion

Understanding supervised machine learning models by carefully curating, controlling and modifying the data flow into them allows for improved understanding of the results in relation to key variables of interest. If the changes observed do not make scientific sense, then these models and related databases need to be more carefully defined and applied, typically by narrowing the set of variables considered, or stratifying on secondary variables.

Understanding requires careful definition and modification of the data flows upon which the ANN and other models are based. If stratification on the levels of secondary variables does not affect the accuracy of predictive classification, then such variables can be left out of the modeling process.

The inductive nature of A.I. and more restricted predictive supervised ANNs and other models forces researchers to be aware of the data-centric nature of these approaches to modeling and prediction. Even more so than statistical approaches based on population-related probability models, machine learning models require an awareness of issues related to data-related bias, the stability of inferences and how results can be made more generalizable.

Even though machine learning models are complex, the first-stage adjustment strategies outlined here reflect much previous work and allow for insight into the general contours of the underlying dataset to provide greater understanding of the overall results. Applications of these strategies to, among others, infectious disease predictive classification modeling and specific hospital utilization databases issues, are the subject of future work.

## 7. Conclusions

To summarize, a set of data modification and curation strategies available to help better understand the behavior and predictions of ANN and random forest models may include the following:

1. First-stage propensity score adjustment and the use of residuals in the fitting of the ANN model, residualized ANN model fits data having less variation, and thus being more interpretable. These approaches provide greater comparability with previous statistical and mathematical models and greater comparability between ANN models applied to similar datasets drawn from different sites (for example, hospitals or various data collection sites).
2. Use of principal components to orthogonalize the data and aid convergence, comparison of ANN models: stability/convergence and predictive classification accuracy.
3. Combinations of these approaches.
4. Applying the original ANN model within data categories defined by the secondary, mediating variables and examining the stability of the resulting predictive classification may be necessary.
5. The use of localized windows across the set of variables, and the stability of the resulting data patterns and machine learning model across the many possible subsets of secondary variables (LASSO type restrictions) and across demographic sub-groups.
6. Propensity score-based stratification of the data.
7. Modification of predictive classification thresholds in the testing dataset.
8. Censoring issues for specific types of data.
9. Data structures and possible bias issues in the data.
10. The need to carefully define data variables and their related numerical scales.

## Figures and Tables

**Table 1:** Predictive classification comparison.

Sensitivity	Specificity	False +	False −	Accuracy	AUC
Logistic Regression (classification threshold = 0.5)					
14.3%	98.9%	1.2%	85.7%	92.6%	86.7%
ANN (hidden layers = 2, classification threshold = 0.5)					
33.3%	93.6%	6.4%	66.6%	89.3%	72.4%
ANN (hidden layers = 12, classification threshold = 0.5)					
40.0%	97.3%	2.8%	60.0%	90.5%	70.5%

## Data Availability

Data available upon request.
